# Bioinformatical analysis and experimental validation of endoplasmic reticulum stress-related biomarker genes in type 2 diabetes mellitus

**DOI:** 10.3389/fgene.2024.1445033

**Published:** 2024-11-01

**Authors:** Lili Yao, Jie Xu, Xu Zhang, Zhuqi Tang, Yuqing Chen, Xiaoyu Liu, Xuchu Duan

**Affiliations:** ^1^ Key Laboratory of Neuroregeneration of Jiangsu and Ministry of Education, Nantong Laboratory of Development and Diseases, Department of Endocrine, Department of Pharmacy, School of Life Science, Co-innovation Center of Neuroregeneration, Medical School, Affiliated Hospital of Nantong University, Nantong University, Nantong, China; ^2^ Clinical Medical Research Center, Wuxi No. 2 People’s Hospital, Jiangnan University Medical Center, Wuxi, China

**Keywords:** type 2 diabetes mellitus, biomarker gene, endoplasmic reticulum stress, bioinformatical analysis, experimental validation, diagnosis

## Abstract

**Introduction:**

Endoplasmic reticulum stress (ERS) is a prominent etiological factor in the pathogenesis of diabetes. Nevertheless, the mechanisms through which ERS contributes to the development of diabetes remain elusive.

**Methods:**

Transcriptional expression profiles from the Gene Expression Omnibus (GEO) datasets were analyzed and compared to obtain the differentially expressed genes (DEGs) in T2DM. Following the intersection with ERS associated genes, the ERS related T2DM DEGs were identified. Receiver operating characteristic (ROC) and Least Absolute Shrinkage and Selection Operator (LASSO) analysis were performed to screen out the ERS related biomarker genes and validate their diagnostic values. Gene expression level was detected by qPCR and Elisa assays in diabetic mice and patient serum samples.

**Results:**

By analyzing the transcriptional expression profiles of the GEO datasets, 49 T2DM-related DEGs were screened out in diabetic islets. RTN1, CLGN, PCSK1, IAPP, ILF2, IMPA1, CCDC47, and PTGES3 were identified as ERS-related DEGs in T2DM, which were revealed to be involved in protein folding, membrane composition, and metabolism regulation. ROC and LASSO analysis further screened out CLGN, ILF2, and IMPA1 as biomarker genes with high value and reliability for diagnostic purposes. These three genes were then demonstrated to be targeted by the transcription factors and miRNAs, including CEBPA, CEBPB, miR-197-5p, miR-6133, and others. Among these miRNAs, the expression of miR-197-5p, miR-320c, miR-1296-3P and miR-6133 was down-regulated, while that of miR-4462, miR-4476-5P and miR-7851-3P was up-regulated in diabetic samples. Small molecular drugs, including D002994, D001564, and others, were predicted to target these genes potentially. qPCR and Elisa analysis both validated the same expression alteration trend of the ERS-related biomarker genes in diabetic mice and T2DM patients.

**Discussion:**

These findings will offer innovative perspectives for clinical diagnosis and treatment strategies for T2DM.

## Introduction

Type 2 diabetes mellitus (T2DM) is presently recognized as the third most severe chronic ailment globally, following cancer and cardiovascular disease, posing a significant threat to human wellbeing (Chen et al., 2012). The pathogenesis of T2DM encompasses intricate mechanisms, including immune dysfunction (Geerlings and Hoepelman, 1999), oxidative stress (Darenskaya et al., 2021), mitochondrial dysfunction (Wada and Nakatsuka, 2016), glucose toxicity (Robertson and Harmon, 2006) and endoplasmic reticulum stress (ERS) (Lee and Lee, 2022). The complexities of diabetic complications, such as diabetic neuropathy and nephropathy, pose challenges in the development of efficacious medications. However, due to the limited understanding of the pathogenesis, clinically, there are no reliable biomarkers for early detection of diabetes, and treatment continues to be challenging.

The endoplasmic reticulum (ER), one of the most extensive organelles within cells, possesses a vast membrane structure and serves as the site for initial protein synthesis and folding. The disruption of internal environmental stability in the endoplasmic reticulum, caused by the stimulation of factors such as ion storage and lipid synthesis, can lead to protein misfolding, referred to as ERS (Cnop et al., 2017). Excessive ERS can impede cellular function, resulting in aberrant protein synthesis and degradation, cellular oxidation, apoptosis, inflammatory responses, and the onset of various diseases (Zhang et al., 2022). ERS plays a pivotal role in the pathogenesis and progression of numerous disorders, including diabetes, Alzheimer’s disease, Parkinson’s disease, and other related conditions (Ghemrawi and Khair, 2020).

Previous studies have been conducted to explore the interplay between ER stress and diabetes and its complications. A prior study has elucidated the involvement of the IP3R1-GRP75-VDAC1 complex in mediating ER stress and mitochondrial oxidative stress and its significant role in atrial remodeling in diabetes (Yuan et al., 2022). Additionally, it has been reported that in diabetic mice, ER stress and autophagy play a regulatory role in neuronal survival and death, with the ER stress pathway potentially contributing to diabetes-induced neurotoxicity and cognitive impairment (Kong et al., 2018). These findings suggested the vital roles of ERS in the occurrence and progression of diabetes and its complications.

T2DM is primarily caused by decreased pancreatic β cells and insulin secretion (Eizirik et al., 2020; Sun et al., 2023). ERS has been considered a critical contributing factor to unfolded protein response (UPR) and the dysfunction of β cells, which is essential factor T2DM pathogenesis (Yong et al., 2021; Sak et al., 2024; Zhang et al., 2023). To explore the critical ERS-related biomarker genes in T2DM, we investigated the association between ERS-related genes and the differentially expressed genes (DEGs) in islets of T2DM patients through bioinformatical analysis. The GEO dataset GSE25724 was employed for Gene Set Enrichment Analysis (GSEA) and for identifying DEGs in T2DM. Following the intersection with the ERS-related genes, T2DM-associated ERS-DEGs were obtained. Three critical biomarker genes were further screened and validated by Receiver Operating Characteristic (ROC) and Least Absolute Shrinkage and Selection Operator (LASSO) analysis. Ultimately, the relative expression levels of the critical biomarker genes were determined using qPCR on constructed T2DM mice and T2DM patients. The findings will help to further understand the pathogenesis and provide novel insights into the clinical diagnosis and treatments of T2DM.

## Materials and methods

### Data collection

The dataset GSE25724, which contains the transcriptional expression profiles of normal and diabetic tissue samples, was obtained from the Gene Expression Omnibus (GEO) database (http://www.ncbi.nlm.nih.gov/geo/) and used for the analysis of DEGs in T2DM. The expression profile analysis was conducted on the GPL96 platform, and the sequencing was performed using Affymetrix Human Genome U133A Array technology. The subjects included seven healthy human islet tissue samples and six islet tissue samples from patients with type II diabetes. GSE118139 and GSE20966, which contain two control islet tissue samples and two diabetic samples, ten control islet samples, and ten diabetic samples, respectively, were used for LASSO analysis to validate and select the biomarker genes. GSE15932, GSE15653, GSE166467, GSE55650, and GSE20966 were used to validate the expression levels of the biomarker genes. GSE15932 contains peripheral blood samples of eight patients with T2DM and eight healthy controls. The GSE15653 dataset consisted of 13 obese (9 with T2DM) and five control subjects from human surgical liver biopsies. GSE166467 comprised the mRNA expression data for both proliferating myoblasts and differentiated myotubes from 13 T2DM patients and 13 controls. GSE55650 consisted of the muscle biopsies from 6 T2DM patients and six controls.

The human genes related to ERS were collected by combining the genes from the GeneCards database (GeneCards; https://www.genecards.org/) (970 protein genes) and a list from the literature (26 protein genes) (Shen et al., 2022). After the intersection, 973 ERS-related protein genes were obtained and used for the subsequent analysis.

### Screening of differentially expressed ERS-related genes

Based on the expression data provided by the dataset GSE25724, DEGs between normal and diabetic islet samples were analyzed. Firstly, batch effects were excluded by principal component analysis (PCA). The “limma” package of R software was used to identify the DEGs in diabetes. With the absolute value | log2 FC |>2, the genes with significant expression differences were selected after calibration for *p* < 0.05. The heatmap and volcano plots of the DEGs were generated using the “heatmap” and “ggplot2″ packages of R software. The intersection of the ERS-related genes and the DEGs from GSE25724 is considered the ERS-related DEGs in diabetes.

### Protein-protein interaction networks analysis

The Protein-Protein Interaction (PPI) network analysis was conducted using the Search Tool (STRING) database. A composite score of >0.4 was considered statistically significant. The analysis results were visualized using Cytoscape software (version 3.8.1). The Spearman correlation of candidate genes was analyzed using R software’s “coreplot” package.

### Functional enrichment analysis

GO and KEGG pathway enrichment analysis was conducted using the “GO plot” package of R software to predict the potential molecular functions of the biomarker genes. The GO settings include molecular function (MF), biological process (BP), and cellular composition (CC). GSEA analysis was conducted using the Xiantao Academic Analysis Platform (https://www.xiantaozi.com) to find the pathways to enrich the DEGs.

### Validation of the ERS-related DEGs

Receiver operating characteristic (ROC) curves were performed using the “pROC” package to evaluate the reliability of the ERS-related DEGs. Subsequently, the genes selected by ROC were further screened by Least Absolute Shrinkage and Selection Operator (LASSO) Cox analysis using the datasets GSE25724, GSE118139, and GSE20966 to give out the critical biomarker genes. The biomarker genes for predicting were then analyzed in the validation sets, including GSE118139, GSE15932, GSE15653, GSE166467, GSE20966, and GSE55650.

### Prediction of transcription factors and microRNAs

The transcription factors regulating the biomarker genes were predicted by the “hTFtarget” database (http://bioinfo.life.hust.edu.cn/hTFtarget#!/). The gene transcription factor network diagram was generated by “Cytoscape.” MiRNAs targeting the biomarker genes were predicted in miRWalk (http://mirwalk.umm.uni-heidelberg.de/) and miRTarBase (https://mirtarbase.cuhk.edu.cn/∼miRTarBase/miRTarBase_2022/php/index.php), by setting the conditions for “number_of_pairings>15, binding_region_length>20 and longest_consecutive_pairings>10”.

### Drug analysis of the biomarker genes

Potential drugs with CAS numbers interacting with the biomarker genes were predicted by the CTD online tool (https://ctdbase.org/). The drugs were screened using the website’s scores. The top 20 drugs for each biomarker gene were collected.

### Construction of diabetic mouse model

Forty 6-week-old wild-type B6 male mice (weighing about 18–20 g) were randomly divided into the control group and the model group. The model group was fed a high-fat diet (45% fat content) for 6 weeks and treated by intraperitoneal injection of 30 mg/kg streptozotocin once. The type 2 diabetic mice were constructed when the Fasting blood glucose was higher than 7.8 mmol/L, and the random blood glucose was higher than 16.7 mmol/L. All animal protocols were approved by the Committee of Nantong University (SYXK (SU) 2017-0046) and the Administration Committee of Experimental Animals, Jiangsu Province, China.

### Human samples collection

For qPCR detection of the biomarker genes, eight blood samples were collected from the Department of Endocrinology, Affiliated Hospital of Nantong University, containing 4 T2DM samples and four non-related control samples. For qPCR detection of the miRNAs and Elisa detection of the biomarker genes, another 12 blood samples (6 T2DM and six non-related control samples) were collected from the same hospital department. The case information of all the samples is listed in (Supplementary Table. 1, 2). After overnight fasting, 5 mL venous blood samples were collected and centrifuged at 3,000 *g* for 10 min, followed by serum separation and storage at −80°C. The study and experiments were approved by the Administration Committee of Nantong University Affiliated Hospital (2018-K016), Jiangsu Province, China. All volunteers involved in this study provided written consent for publication.

### RNA extraction and qPCR analysis

For detecting the expression of the critical ERS-related DEGs, the total RNA of diabetic and normal islet tissues of mice and human serum plasma samples was isolated with Trizol (Invitrogen) and stored at −80°C. The cDNA was synthesized using the Transcriptor First Strand cDNA Synthesis Kit (Roche) according to the manufacturer’s instructions and stored at −20°C. The qPCR reaction was performed in triplicates using the FastStart Universal SYBR Green Master Mix (Roche Applied Science) on a real-time PCR detection system (StepOneTM Real-Time PCR Systems). The primers of the critical ERS-related DEGs and reference gene (18S) for qPCR were designed by Beacon Designer eight and the sequences are listed below (Table 1):

For miRNA expression analysis, all small RNAs were extracted from serum plasma by using mirVana™ miRNA isolation kit (ThermoFisher). The reverse transcription and qPCR reaction were performed using miRNA first Strand cDNA Synthesis Kit (by stem-loop) and miRNA Universal SYBR qPCR Master Mix (Vazyme), respectively. While the reverse universal primer is provided by the Universal SYBR qPCR Master Mix, the specific forward primers for miRNAs’ qPCR detection were designed according to the manufacturer’s instruction and listed below (Table 2):

**TABLE 1:** The qPCR primers of the ERS-related biomarker genes.

Genes	Forward (5′-3′)	Reverse (5′-3′)
CLGN-mouse	AGT​GGT​AAT​GTC​TGA​GCA​A	AGG​AGT​TTG​TAG​TGA​TGT​TTG
CLGN-human	TAT​ATG​ACC​CAC​ATT​TAC​CTA​GT	AAC​CCA​TTA​TCC​TTG​TAT​TCA​AA
IMPA1-mouse	AGA​ATT​GGA​ATC​GGA​CAG​A	CAA​GTT​TAG​ATC​AGT​GGA​TAG​C
IMPA1-human	TTG​CCT​GTA​ATC​TTT​CCA​AC	TCT​AAG​AAG​TCC​TGT​TAC​TCA​A
ILF2-mouse	TGG​CTT​CTA​TAA​CCT​CAG​TAG	GCT​TTC​ACC​CCA​CAT​TTA​G
ILF2-human	GTAGGGCTCTTGGTCTTT	AGGTTCCAGGAGTTTGTC

**TABLE 2:** The specific qPCR primers of the miRNAs predicted to target the ERS-related biomarker genes.

Genes	Forward (5′-3′)
miR-197–5p	CGC​GGG​TAG​AGA​GGG​CAG​T
miR-6133	CGCGTGAGGGAGGAGGT
miR-7851–3p	CGGAGTGGGGCTTCGACC
miR-1296–3p	CGGAGTGGGGCTTCGACC
miR-320c	CGCGAAAAGCTGGGTTGA
miR-4776–5p	CGGTGGACCAGGATGGCA
miR-4462	GTGACACGGAGGGTGGCT
MiR-16–5p (reference)	CGC​GTA​GCA​GCA​CGT​AAA​TA

### Statistical analysis

**FIGURE 1:**
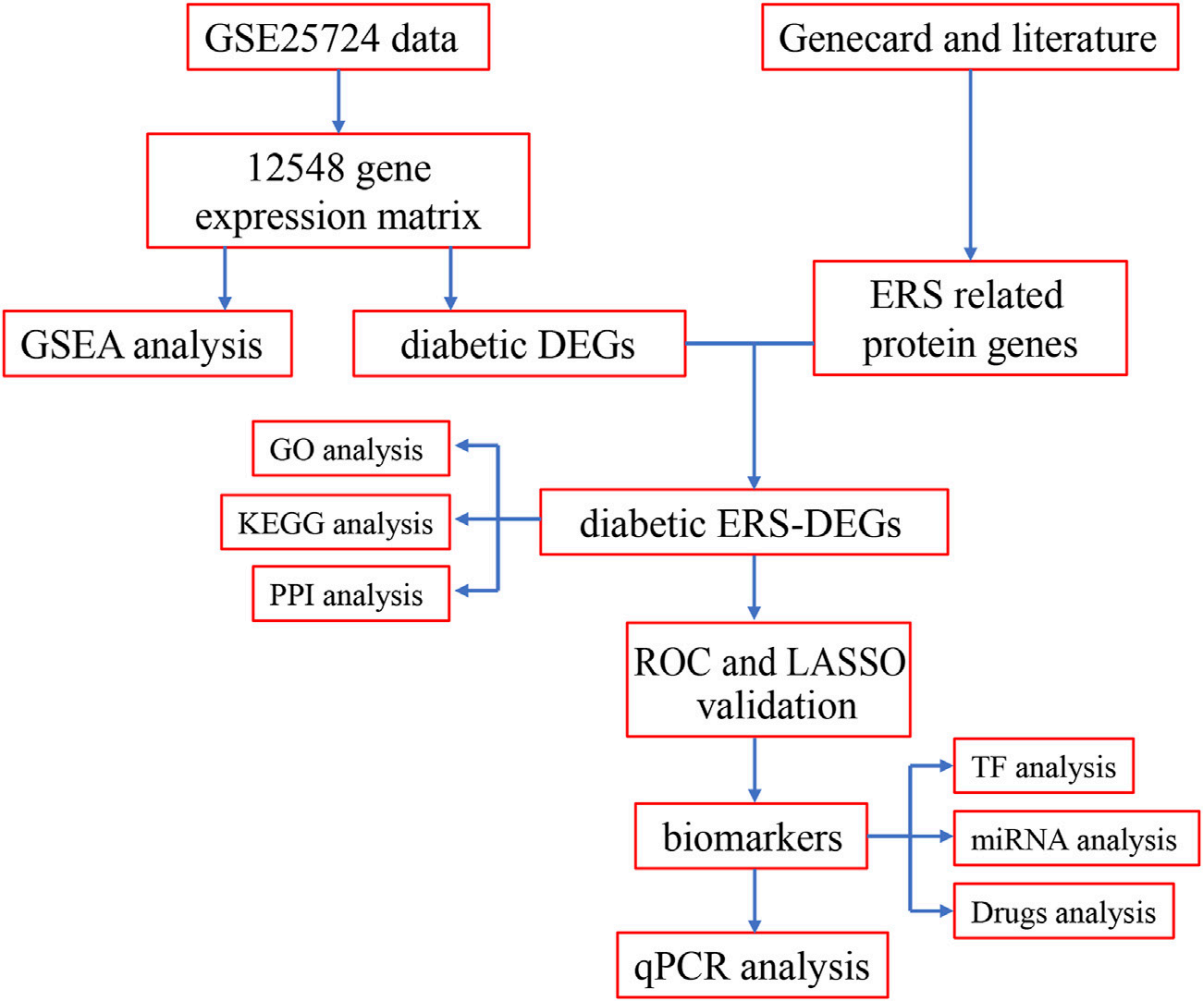
Flow chart of methodologies applied in the study.

R software (version 3.6.2) was utilized for statistical analysis. Student’s t-test was used to evaluate the significance of variance. A *p*-value of <0.05 was considered statistically significant. The overview of the workflow is shown in Figure 1.

## Results

### Identification of ERS-related DEGs in diabetes

**FIGURE 2:**
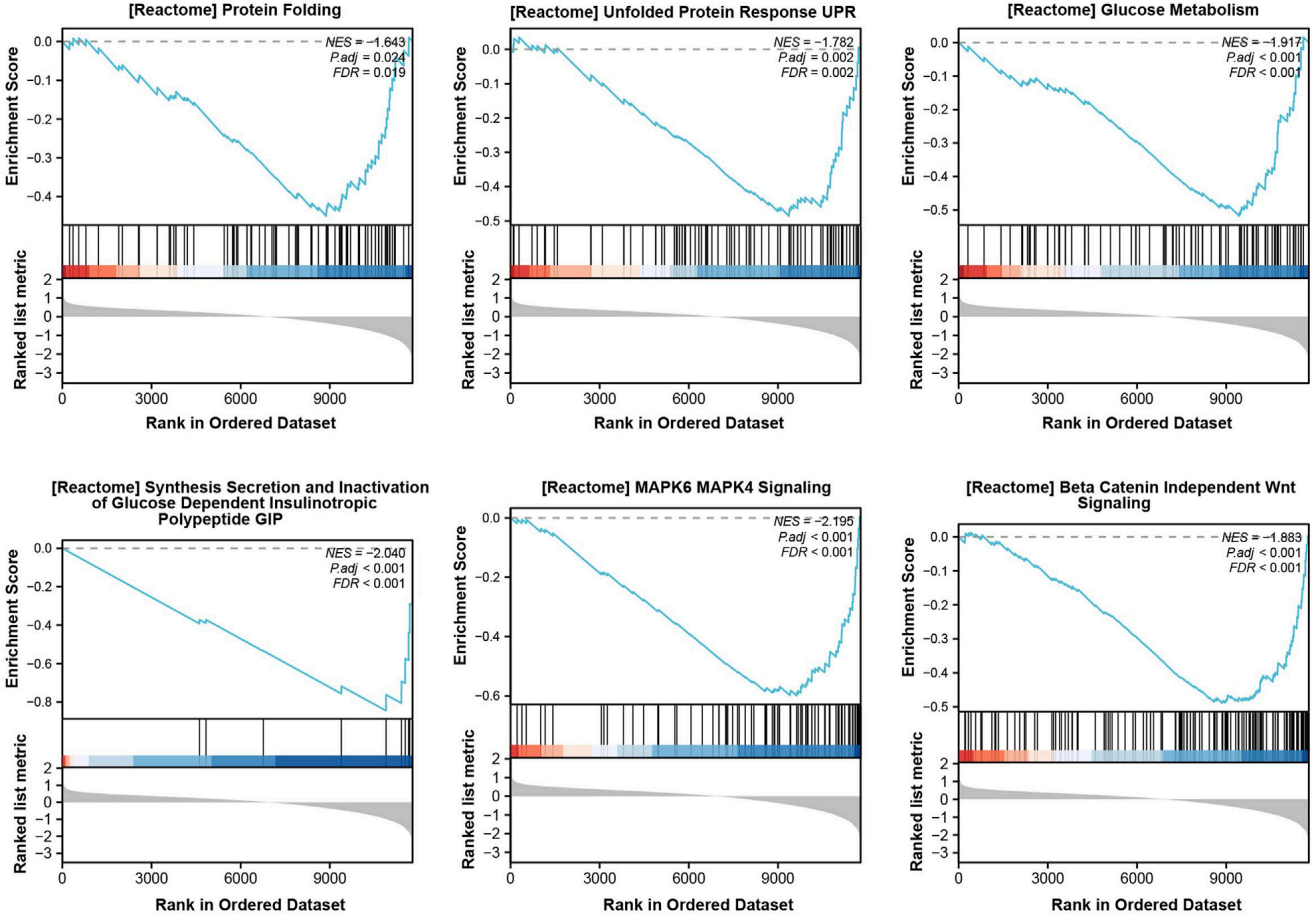
GSEA enrichment analysis for T2DM-related pathways.

**FIGURE 3:**
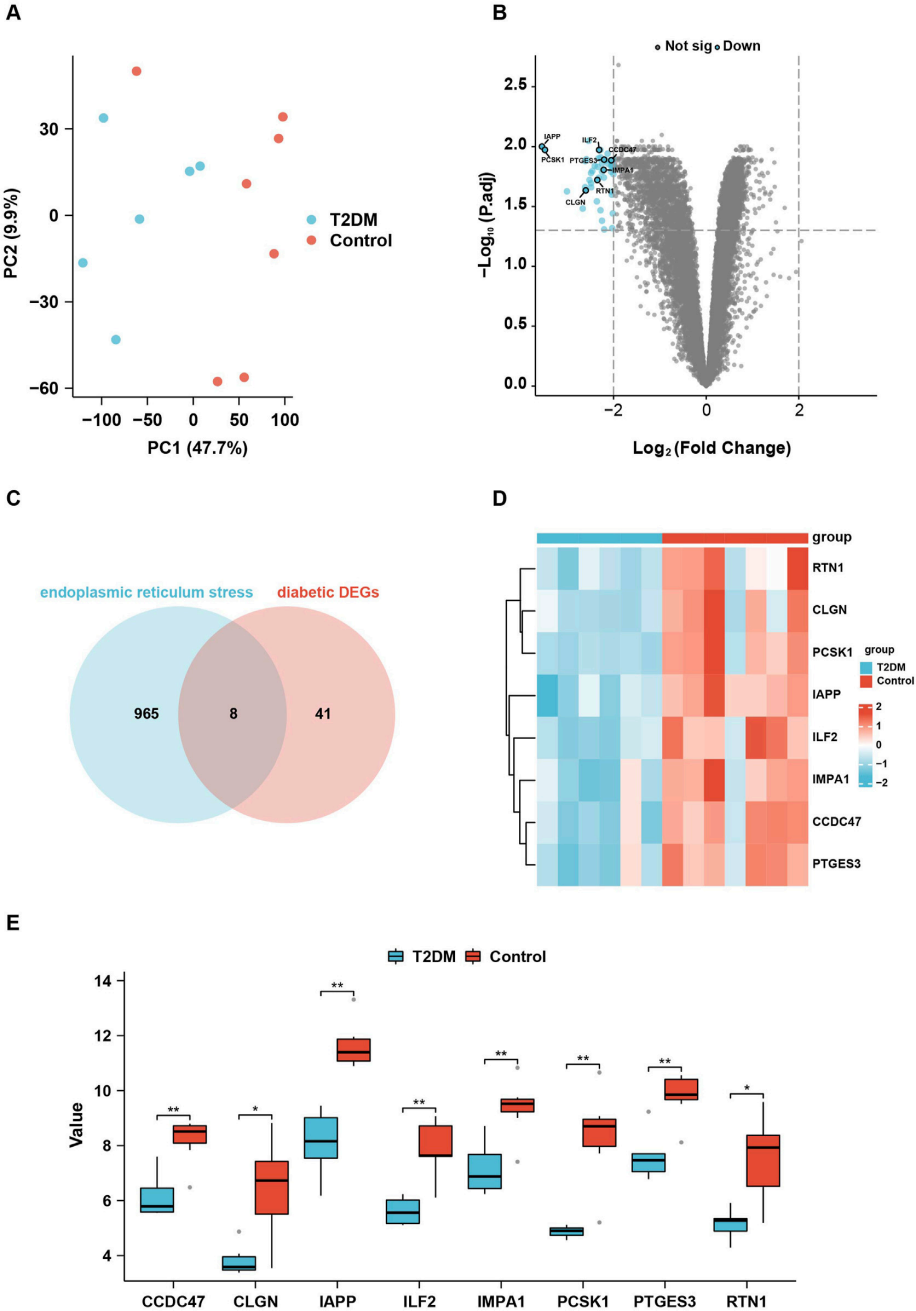
Identification of the ERS-related DEGs in T2DM. **(A)**, PCA analysis of the gene expression data from GSE25724. **(B)**, the volcano plot showing the DEGs in T2DM. **(C)**, the Venn diagram showing the intersection between the DEGs in T2DM and the ERS-related genes. **(D, E)** *,* the heatmap and box plots showing the eight relative expression levels of ERS-related DEGs in T2DM.

From the dataset, GSE25724 yielded 12,548 genes found to be diabetes-related protein genes. In order to investigate the pathways associated with diabetes, Gene Set Enrichment Analysis (GSEA) was conducted on these 12,548 genes. The findings revealed a significant enrichment of the “Unfolded Protein Response” (UPR) pathway, which demonstrated a close correlation with T2DM, thereby suggesting the crucial involvement of ERS in the development of T2DM (Figure 2). Principal Component Analysis (PCA) was employed to validate the reproducibility and reliability of the data obtained from GSE25724 (Figure 3A). Subsequently, the gene expression profiles of normal and diabetic islet tissues in GSE25724 were analyzed for selecting the DEGs related to ERS, with the screening criteria of |logFC| >2 and *p* < 0.05. As a result, 49 protein genes were identified as T2DM-associated DEGs, which were visualized in a volcano plot (Figure 3B). After intersection with the 973 ERS related genes, 8 ERS-related DEGs (RTN1, CLGN, PCSK1, IAPP, ILF2, IMPA1, CCDC47, and PTGES3) were screened out as T2DM related ERS-DEGs, which were indicated to be downregulated in diabetic samples (Figures 3C–E).

### Functional enrichment and PPI network analysis

**FIGURE 4:**
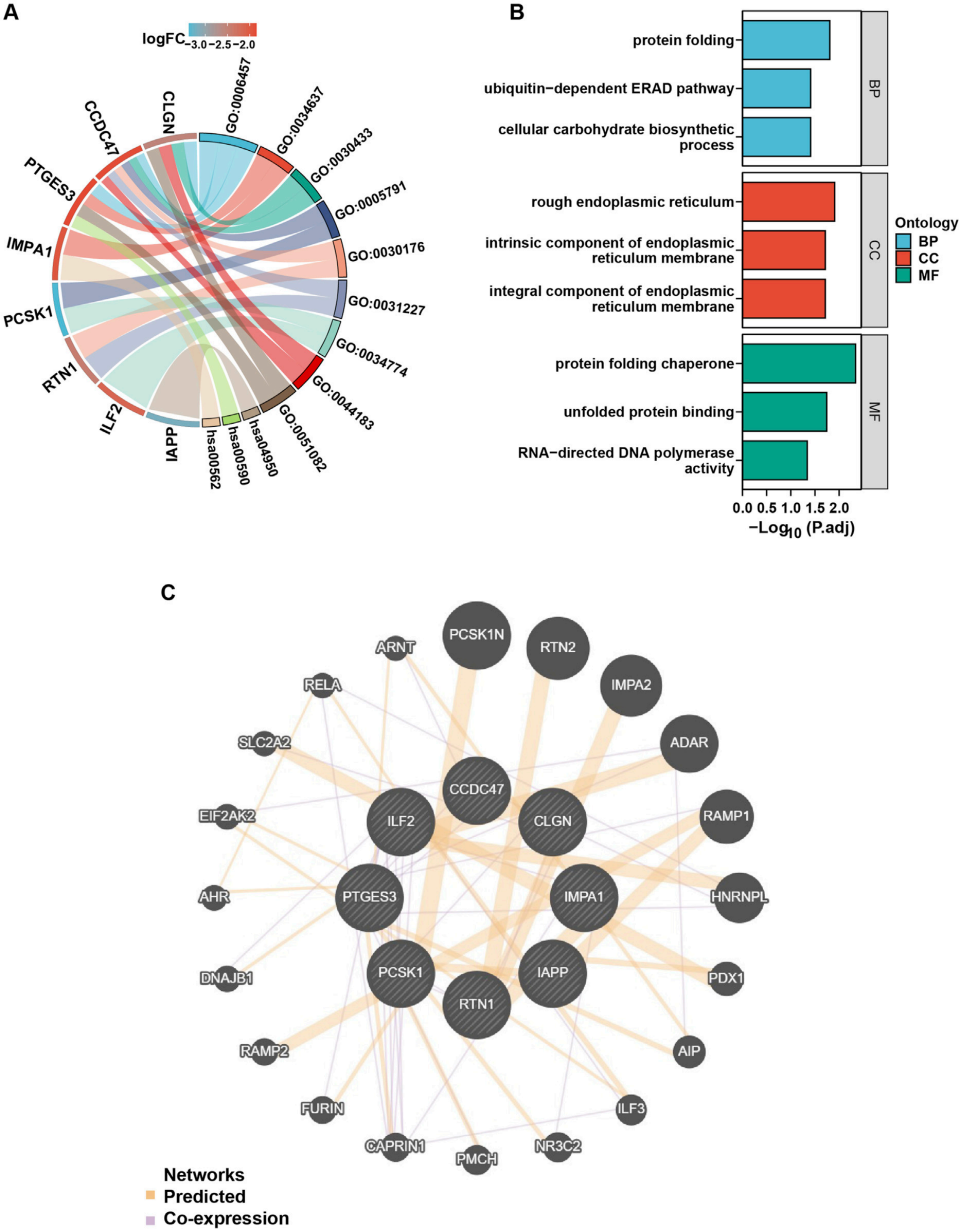
GO, KEGG, and PPI analysis of the ERS-related DEGs in T2DM. **(A)** *,* the chord diagram of GO analysis on the ERS-related DEGs. GO: 0006457 = protein folding, GO: 0034637 = cellular carbohydrate biosynthetic process, GO: 0030433 = ubiquitin-dependent ERAD pathway, GO: 0005791 = rough endoplasmic reticulum, GO: 0030176 = integral component of endoplasmic reticulum membrane, GO: 0031227 = intrinsic component of endoplasmic reticulum membrane, GO: 0034774 = secretory granule lumen, GO: 0044183 = protein folding chaperone, GO: 0051082: unfolded protein binding, hsa00562 = inositol phosphate metabolism, hsa00590 = arachidonic acid metabolism, hsa04950 = maturity onset diabetes of the young, p. adjust value < 0.05. **(B)** *,* GO enrichment map with ERS-related DEGs, p. adjust value < 0.05. **(C)** *,* PPI network analysis of the ERS-related DEGs.

In order to gain further insight into the functions and pathways associated with the eight genes selected above, GO and KEGG enrichment analyses were conducted. The findings revealed that these genes are primarily involved in protein folding, the ubiquitin-dependent ERAD pathway, and cellular carbohydrate biosynthetic metabolism. The protein products of the genes are primarily localized on the rough endoplasmic reticulum, the intrinsic component of the endoplasmic reticulum membrane, and the integral component of the endoplasmic reticulum membrane. These proteins are involved in protein folding chaperones, unfolded protein binding, and RNA-directed DNA polymerase activity (Figures 4A, B).

PPI network analysis was conducted using the online tools “GeneMANIA” and “Cytoscape” software to investigate the interaction between the eight candidate genes and other protein genes. The results revealed strong associations between the genes. For example, CAPRIN and PCSK1 were co-expressed, and RELA was co-expressed with CCDC47 and PTGES3 (Figure 4C).

### Screening and validation of the biomarker genes for T2DM

**FIGURE 5:**
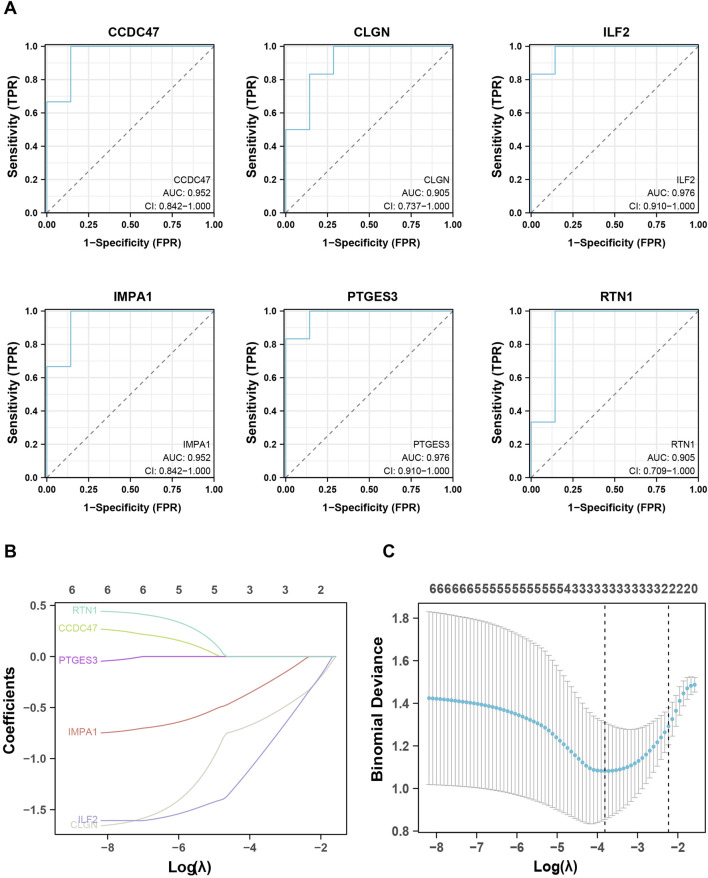
Identification and validation of the biomarker genes for diagnosis. **(A)** *,* ROC analysis shows the six critical genes with AUC values higher than 0.9. **(B, C)** *,* LASSO analysis screened out the three biomarker genes.

To evaluate the predictive and diagnostic value of the ERS-related genes in T2DM, the Receiver Operating Characteristic (ROC) curves were employed to assess the diagnostic efficacy of the eight genes selected above. The findings revealed that six genes (CCDC47, CLGN, ILF 2, IMPA 1, PTGES3, RTN 1) exhibited an area under the curves (AUC) exceeding 0.9, indicating a substantial diagnostic value (Figure 5A).

**FIGURE 6:**
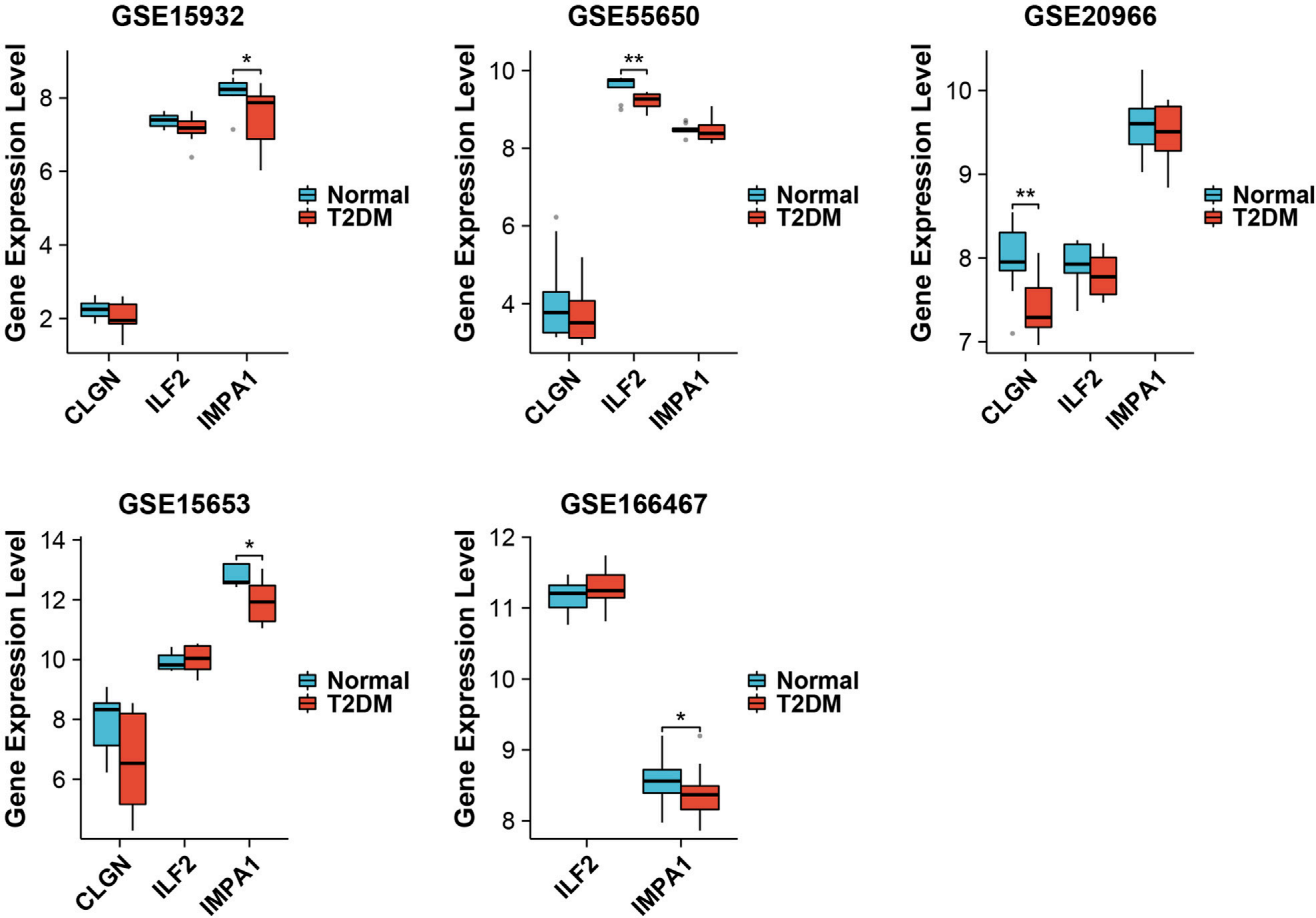
Validation of the biomarker genes in other datasets. “*” represents *p* < 0.05, “**” represents *p* < 0.01.

To further observe and evaluate the correlation of these six genes with T2DM, LASSO regression analysis was executed based on the gene expression databases GSE25724, GSE118139, and GSE20966, wherein CLGN, ILF2 and IMPA1 were identified as critical biomarker genes for T2DM and therefore used for subsequent analysis (Figures 5B, C). In addition, the expression levels of these three genes were examined in other validation datasets. As a result, IMPA1 was found to be significantly downregulated in the diabetes group in databases GSE15932, GSE15653, and GSE166467. CLGN and ILF2 were significantly downregulated in the diabetic group in GSE20966 and GSE55650, respectively (Figure 6).

### Transcription factor analysis

**FIGURE 7:**
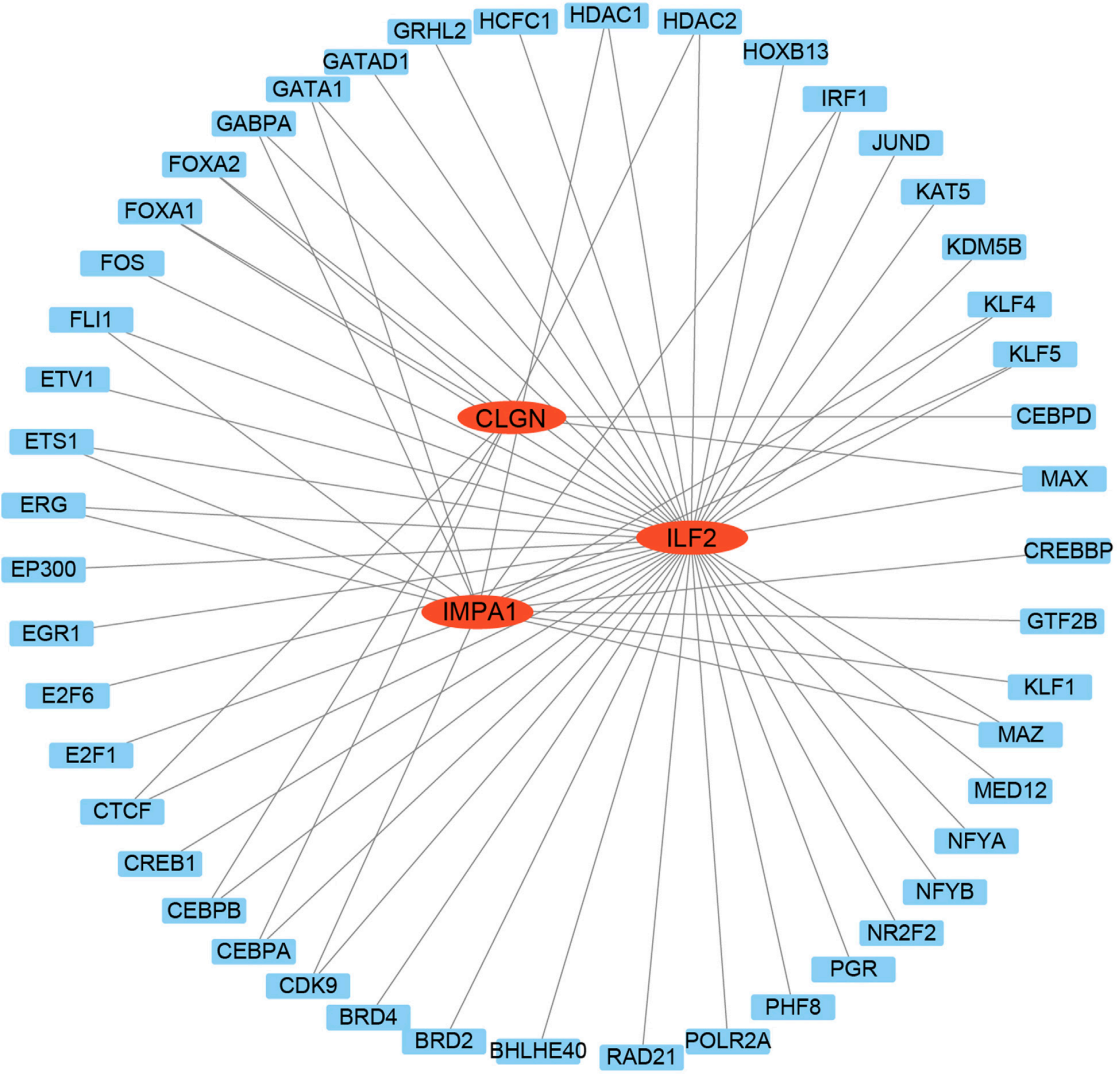
Transcription factors analysis of the biomarker genes. The TFs were used as nodes in an interconnected regulatory network. Red ellipses represent the biomarker genes, and blue rectangles represent the TFs.

To further elucidate the upstream regulators of the biomarker genes, a transcription factor network analysis was performed to investigate the transcription factors regulating the biomarker genes with significant diagnostic potential. The findings revealed that multiple transcription factors potentially regulate most of these genes. For instance, CLGN was predicted to be targeted by FOXA1, FOXA2, CEBPA, CEBPB, and others. E2F1, CDK9, MAZ, KLF1, and others can target IMPA1. ILF2 might be targeted by MAX, KLF5, KLF4, JUND, and so on (Figure 7). The same transcription factors, such as HDAC1, HDAC2, CEBPA, and CEBPB could also regulate different biomarker genes.

### miRNA analysis

**FIGURE 8:**
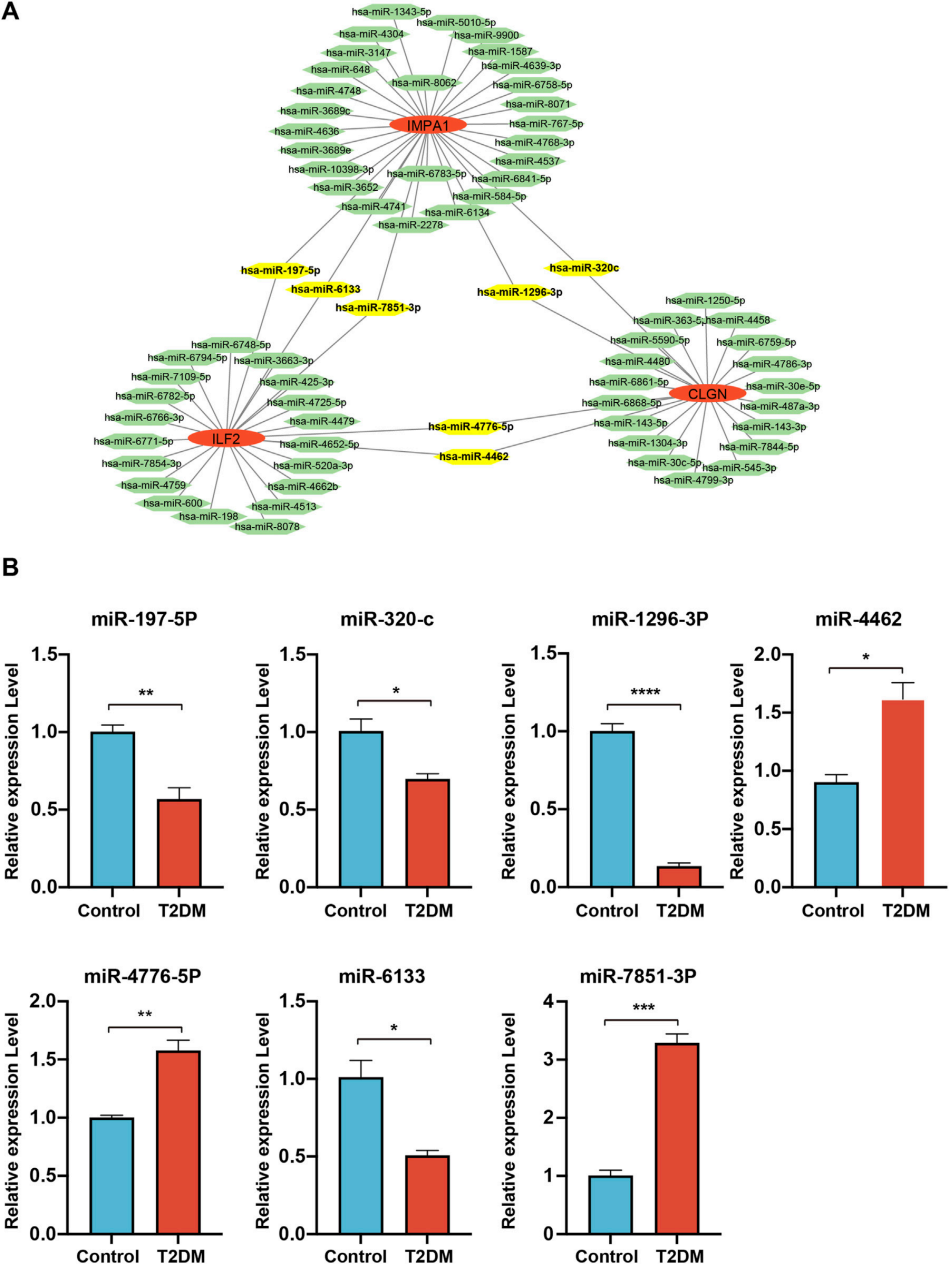
miRNA analysis of the biomarker genes. **(A)** *,* the miRNAs are predicted to target the biomarker genes. MiRNAs are represented by green hexagons. **(B)** *,* qPCR detection of the expression levels of the miRNAs simultaneously targeting two of the biomarker genes.

To understand the potential roles of miRNAs involved in regulating these three biomarker genes, miRWalk and miRTarBase were utilized for microRNA prediction. Numerous miRNAs were predicted to be potential upstream regulators of the biomarker genes. Among them, hsa-miR-197–5p, hsa-miR-6133, hsa-miR-7851–3p, hsa-miR-1296–3p, hsa-miR-320c, hsa-miR-4776–5p and hsa-miR-4462 were predicted to simultaneously target two of the biomarker genes (Figure 8A). We then performed qPCR analysis to detect the relative expression levels of these miRNAs in serum samples, which were revealed to be all significantly changed in diabetic patients. Among them, the expression of miR-197–5p, miR-320c, miR-1296–3P and miR-6133 was downregulated, while that of miR-4462, miR-4476–5P and miR-7851–3P was upregulated in diabetic samples (Figure 8B).

### Drug identification and selection

**FIGURE 9:**
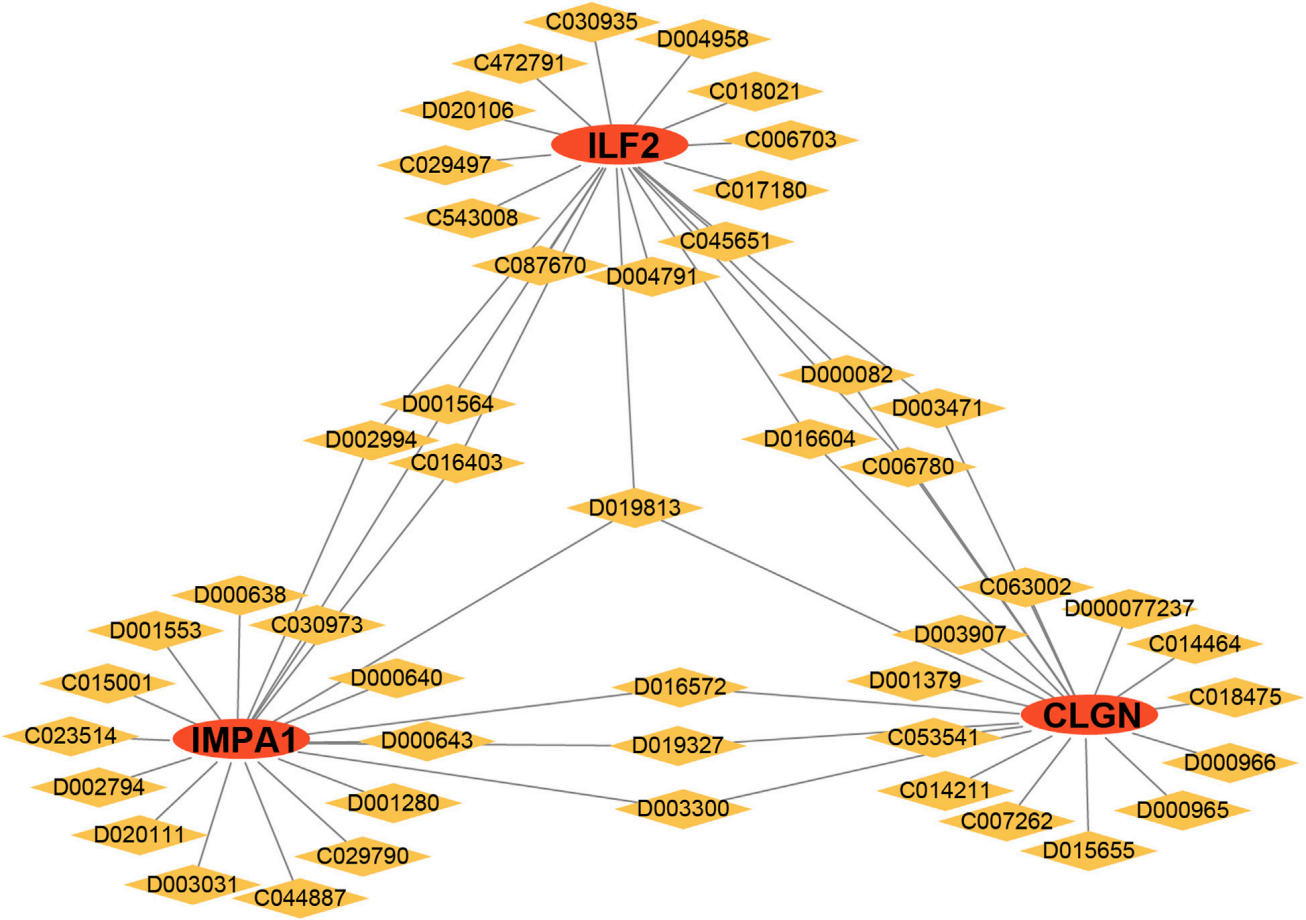
Drug analysis of the biomarker genes. Red ellipses represent the biomarker genes, and yellow diamonds represent the drug IDs.

To identify personalized medicines for diabetes, the CTD website was utilized to predict small molecular drugs. Based on the website’s scores, the top 20 drugs for each gene were selected and presented herein. Interestingly, D019813 (1, 2-Dimethylhydrazine) was predicted to target all three biomarker genes. At the same time, several other drugs, such as D002994 (Clofibrate), D001564 (Benzo(a)pyrene), C016403 (2, 4-dinitrotoluene), D016604 (Aflatoxin B1), D000082 (acetaminophen), D003471(Cuprizone), C006780 (bisphenol A), D016572 (Cyclosporine), D019327 (Copper Sulfate) and D003300 (Copper), exhibited the potential to target two biomarker genes simultaneously. The result suggests that these drugs may serve as effective multi-target medications for T2DM (Figure 9).

### Experimental validation of the expression of the ERS-related biomarker genes

**FIGURE 10:**
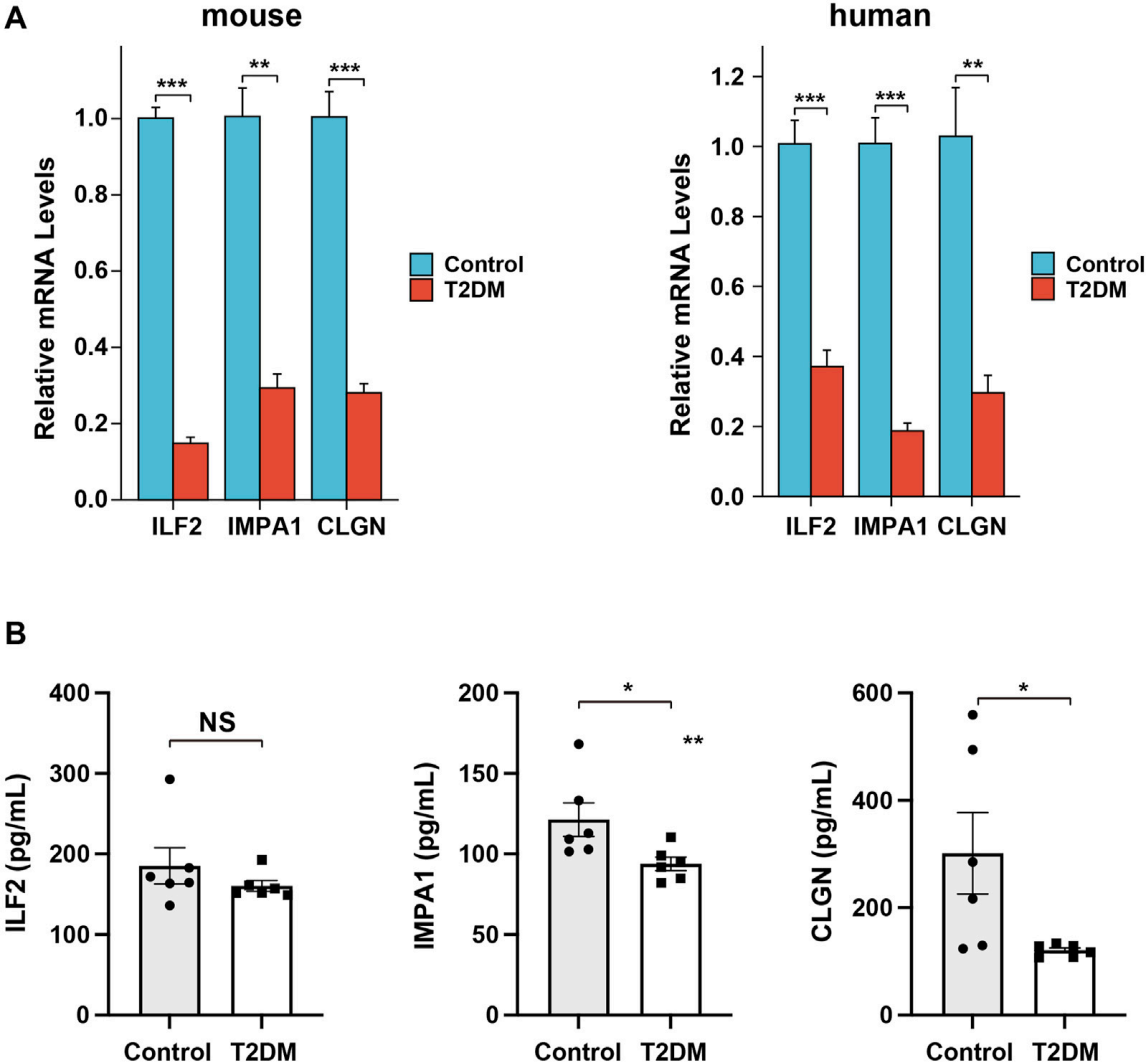
Expression analysis of the biomarker genes in mice and patient serum samples. **(A)** *,* qPCR analysis of the relative expression levels of the biomarker genes in diabetic mice and patient serum samples. **(B)** *,* Elisa analysis of the serum protein levels of the biomarker genes in diabetic and non-related patient samples.

In order to provide additional evidence for the differential expression of the ERS-related biomarker genes in diabetes, we conducted qPCR analysis on the islet tissues of healthy and diabetic mice. The results demonstrated a significant decrease in the transcriptional expression of all three genes in the diabetic samples compared to the control samples (Figure 10A). In addition, to determine whether these biomarker genes can be used for clinical detection, we also performed the qPCR and Elisa analysis on human serum samples of T2DM patients and non-related individuals. The results revealed the same changing trend of gene expression in diabetes, although the alteration of the serum protein level of ILF2 was not significant (Figures 10A, B). The experimental findings aligned with the results obtained from the bioinformatical analysis.

## Discussion

T2DM is a metabolic syndrome characterized by insulin resistance, relative insulin deficiency, and impaired glucose tolerance (Artasensi et al., 2020). The occurrence of ERS is implicated in regulating diverse physiological processes (Jayasooriya et al., 2018), such as inflammation (Zhang et al., 2020), tumor development (Cubillos-Ruiz et al., 2017), anti-viral response (Ong et al., 2018), and lipid metabolism (Zhao et al., 2020). ERS induced by elevated levels of glucose, fat, and cytokine stimulation has been observed to contribute to insulin resistance and the deterioration of islet β-cell function in T2DM (Vallée et al., 2020; Yilmaz, 2017; Deng et al., 2022; Chen et al., 2018). Although numerous studies have demonstrated the association between ERS and T2DM, few studies have been done to explore the utility of the ERS-related biomarker genes for T2DM diagnosis.

In this study, T2DM-associated genes and ERS-associated genes were extracted from the GEO and GeneCards databases, respectively. GSEA analysis of the T2DM-associated genes revealed the enrichment of the diabetic proteins in the pathway of UPR. UPR is crucial in coordinating protein synthesis, folding, and degradation to maintain protein stability, which is vital for cell survival and activity. Prior research has indicated that sustained activation of the UPR plays a role in mitigating ERS-induced disruptions in glucose regulation, chronic inflammation, and the advancement of T2DM (Herrema et al., 2022; Keestra-Gounder et al., 2016; Ma et al., 2014). After the intersection of the two groups of genes, we identified eight ERS-DEGs associated with T2DM, which were all downregulated in the patient samples. These proteins are functionally involved in various biological processes, including cell carbohydrate synthesis, protein folding, inositol phosphatase metabolism, etc. Dysfunctions in the metabolic response to inositol phosphatase have been linked to insulin resistance and the development of long-term microvascular complications in individuals with diabetes (Croze and Soulage, 2013). The concurrent administration of phosphoinositol and inositol has been shown to safeguard hepatocyte integrity and enhance its antioxidant capacity in individuals with T2DM (Foster et al., 2017).

Utilizing ROC and LASSO analysis on the eight ERS-DEGs, three critical genes were subsequently screened out as valuable predictors of the disease, including CLGN, ILF2, and IMPA1. CLGN, known as ER chaperone calmegin, is a highly expressed ER-associated gene in aldosterone-producing adenomas, but its role in diabetes remains unclear (Itcho et al., 2020). ILF2, known as nuclear factor 45 (NF45), is crucial in regulating RNA stability and inflammatory response (Yin et al., 2022). The formation of a complex between ILF2 and S6K protein influences insulin levels and consequently contributes to the progression of metabolic diseases (Das et al., 2018; Pavan et al., 2016). IMPA1 is an enzyme responsible for inositol synthesis; deficiency in IMPA1 leads to a decline in inositol and mitochondrial fission, ultimately leading to the development of diabetes and mitochondrial diseases (Hsu et al., 2021). Recently, a case-control study demonstrated that in gestational diabetes, higher maternal glycemia is associated with decreased protein and mRNA expression levels of IMPA1 (Pillai et al., 2021). In addition, these three genes were further validated in other separated datasets comprising the data from different organs or tissues, including liver, muscle, and peripheral blood of T2DM patients, exhibiting a consistent alteration in expression, thereby suggesting their potential diagnostic value for clinical use.

Our transcription factor analysis identified several crucial transcription factors closely associated with diabetes. Among them, CEBPA and CEBPB are adipogenic transcription factors that not only impact adipogenesis but also influence the occurrence of diabetes (Oh et al., 2013; Wicks et al., 2015). HDAC1 and HDAC2, known as histone deacetylases, are widely recognized for their essential role in regulating DNA structure and gene activity, and they also contribute to the development of diabetic complications (Hou et al., 2018; Zheng et al., 2022; Draney et al., 2018; Li et al., 2022). Additionally, FOXA1 has been demonstrated to play significant roles in maintaining glucose homeostasis and promoting α-cell differentiation (Heddad Masson et al., 2014). In the context of miRNA analysis, it is noteworthy that a few miRNAs exhibit potential targeting capabilities towards two biomarker genes. Our experimental analysis further revealed significant expression alteration of these miRNAs in patient serum samples, suggesting their potential value in diagnosis for T2DM. Among these miRNAs, miR-6133 and miR-320c have been reported to be downregulated in urinary exosomes of T2DM patients in the previous study (Delić et al., 2016), consistent with our results.

Recently, several drugs or biomolecules have been shown to have potential in treating diabetes and its complications via modulating ERS, such as Ghrelin, Rosuvastatin, Selenium Nanodots (SENDs) and Astragalus polysaccharide (Li et al., 2024; Zhao et al., 2023; Huang et al., 2023; Chen et al., 2023). Our study also predicted the drugs having the potential to target the three biomarker genes. Among the top 20 drugs targeting over two genes, cyclosporine has been used for treating T2DM and related complications for a long time (Mahon et al., 1993; Wang et al., 2020). Copper and copper sulfate have been shown to potentially ameliorate diabetes and its complications in animal models (Sitasawad et al., 2001; Sakurai, 2012). However, bisphenol A is a risk factor associated with the occurrence and development of T2DM (Provvisiero et al., 2016). In addition, 1,2-Dimethylhydrazine, Benzo(a)pyrene, and Aflatoxin B1 have been reported to be inducers for cancers including diabetic colon cancer, lung cancer, and hepatocellular carcinoma, suggesting their potentially detrimental effects in application (Terai et al., 2006; Kasala et al., 2015; Cao et al., 2022). The results of the drug analysis indicate that the targeted drugs, which were screened based on the critical diabetic ERS-related DEGs, exhibit significant therapeutic relevance for diabetes treatment. However, it is important to note that certain drugs also present substantial risks, particularly in terms of side effects that may induce carcinogenesis. Therefore, comprehensive evaluation and rigorous testing are imperative during the drug development process.

To further confirm the expression of the biomarker genes, we conducted qPCR experiments using constructed diabetic mice and human serum samples. The results demonstrated a significant decrease in their expression in both diabetic islets and human serum, aligning with the computational analysis. Moreover, the results suggested that these biomarker genes have immense potential in fundamental research and clinical application in T2DM pathogenesis and diagnosis. Comparing with the recent studies which also conducted the bioinformatical analysis of ERS-related biomarkers in T2DM and diabetes nephropathy (Su et al., 2023; Liang et al., 2023), our research has provided more evidence with animal models and clinical samples for the biomarker genes.

There are some limitations of this study. For example, the limited sample size of the T2DM database and the lack of clinical validation. In addition, the mechanism of the critical biomarker genes in regulating T2DM remains unclear. We will continue to delve deeper into the subsequent research about our findings.

## Conclusion

Using bioinformatical methods, the identification and screening of ERS-related genes in diabetes were conducted. Subsequently, three critical biomarker genes were identified and validated through bioinformatical analysis and experimental detection, establishing their utility as biomarkers for T2DM diagnosis. These findings contribute to a deeper comprehension of the interplay between ERS and the onset and progression of diabetes while also offering potential targets for future diagnostic and therapeutic interventions.

## Data Availability

The original contributions presented in the study are included in the article/Supplementary Material, further inquiries can be directed to the corresponding authors.
