# Supplementary material for: Bioinformatical analysis and experimental validation of endoplasmic reticulum stress-related biomarker genes in type 2 diabetes mellitus
Source: Front Genet. 2024 Nov 1;15:1445033. doi: 10.3389/fgene.2024.1445033 (PMC11564187; doi:10.3389/fgene.2024.1445033)
Supplement: Supplementary file 1 [file Table1.docx]

**Supplementary Table 1. Case information for qPCR detection of the three critical genes.**

|  | Age (year) | Gender | Weight (kg) | Fasting glucose (mM) |
| --- | --- | --- | --- | --- |
| Patient 1 | 64 | Male | 65 | 12.3 |
| Patient 2 | 28 | Male | 85 | 12.9 |
| Patient 3 | 53 | Female | 60 | 17.3 |
| Patient 4 | 75 | Female | 58 | 11.2 |
| Non-related 1 | 36 | Male | 90 | 6.4 |
| Non-related 2 | 42 | Female | 47 | 6.8 |
| Non-related 3 | 59 | Male | 75 | 5.9 |
| Non-related 4 | 66 | Female | 55 | 6.1 |

**Supplementary Table 2. Case information for Elisa detection of the three critical genes.**

|  | Age (year) | Gender | Weight (kg) | Fasting glucose (mM) |
| --- | --- | --- | --- | --- |
| Patient 1 | 55 | Female | 61 | 13.1 |
| Patient 2 | 63 | Female | 60 | 9.1 |
| Patient 3 | 56 | Male | 58 | 11.5 |
| Patient 4 | 60 | Female | 60 | 12.8 |
| Patient 5 | 50 | Male | 67 | 10.9 |
| Patient 6 | 49 | Female | 62 | 13.2 |
| Non-related 1 | 35 | Male | 80 | 7.0 |
| Non-related 2 | 47 | Male | 54 | 6.7 |
| Non-related 3 | 41 | Male | 75 | 6.2 |
| Non-related 4 | 68 | Male | 68 | 6.5 |
| Non-related 5 | 42 | Femail | 79 | 5.9 |
| Non-related 6 | 51 | Femail | 65 | 6.1 |
